# Exploring the structure and assembly of seagrass microbial communities in rhizosphere and phyllosphere

**DOI:** 10.1128/aem.02437-24

**Published:** 2025-02-24

**Authors:** Xinqi Li, Hongzhen Wang, Yu Zang, Song Xue, Jiayi Xin, Lei Liu, Xuexi Tang, Jun Chen

**Affiliations:** 1 MoE Key Laboratory of Evolution & Marine Biodiversity, College of Marine Life Sciences, Ocean University of China 506915 , Qingdao, Shandong, China; 2 Key Laboratory of Marine Eco-Environmental Science and Technology, First Institute of Oceanography, Ministry of Natural Resources 99000 , Qingdao, Shandong, China; 3 Laboratory for Marine Ecology and Environmental Science, Qingdao Marine Science and Technology Center, Qingdao, Shandong, China; University of Tennessee at Knoxville, Knoxville, Tennessee, USA

**Keywords:** seagrass, microbial community assembly, rhizosphere, phyllosphere, co-occurrence networks

## Abstract

Microbial community assembly and interactions are pivotal research areas within microbial ecology, yet relevant studies in seagrass rhizospheres and phyllosphere remain relatively scarce. In this study, we utilized high-throughput sequencing technology to investigate the microbial communities in different periods and microhabitats (rhizosphere and phyllosphere) of two seagrass species (*Zostera marina* and *Phyllospadix iwatensis*). Our findings suggest that microhabitats have a more pronounced impact on the composition of seagrass-associated microbial communities compared to periods and species. Further investigations reveal that the phyllosphere microbial community exhibits a more intricate co-occurrence network and interactions than the rhizosphere microbial community. Keystone taxa show distinct functional roles in different microhabitats of seagrasses. Additionally, we observed that differences in seagrass microhabitats influence community assembly, with the rhizosphere microbial community being more influenced by deterministic processes (heterogeneous selection) compared to the phyllosphere. These findings contribute to our understanding of the intricate interactions between seagrasses and their associated microbial communities, providing valuable insights into their distribution patterns and microhabitat preferences.

**IMPORTANCE:**

Studying the community structure and assembly of different microhabitats in seagrass beds contributes to revealing the complexity and dynamic processes of seagrass ecosystems. In the rhizosphere microhabitat of seagrasses, microbial communities may assist in disease resistance or enhance nutrient uptake efficiency in seagrasses. On the other hand, in the microhabitat on the surface of seagrass blades, microorganisms may be closely associated with the physiological functions and nutrient cycling of seagrass blades. Therefore, understanding the structure and assembly mechanisms of rhizosphere and phyllosphere microbial communities is crucial for exploring the interactions between seagrass and microbial communities, as well as for enhancing our comprehension of the stability and resilience of seagrass bed ecosystems.

## INTRODUCTION

Microbial community assembly is a crucial mechanism shaping microbial diversity (1). This process has been extensively studied in soil microbes (2, 3), leaf microbial communities (4), macrofaunal intertidal communities (5), and fragmented seagrass beds (6). To unravel the mechanisms underlying microbial diversity maintenance, researchers have proposed models based on niche, neutral, and process theories, which have been widely applied to investigate microbial community assembly (1, 7–9). Seagrass ecosystems are renowned for their highly diverse habitats and rich microbial communities, providing habitats for a variety of marine organisms and serving numerous ecosystem services (10, 11). These characteristics make seagrass beds an ideal environment for studying microbial community assembly mechanisms (12, 13). Previous research on seagrass ecosystems has characterized the variability of seagrass-associated microbial communities across different habitats and seagrass species or identified core taxonomic shifts driven by environmental changes (14, 15). Additionally, some studies have explored the combined phylogenetic distances among seagrass microbial taxa and the metabolic models of enriched microbial groups to gain deeper insights into the mechanisms underlying seagrass microbiome assembly (16, 17).

Microbial interactions play a pivotal role in maintaining microbial community diversity (18), and correlation-based network analysis has been demonstrated as effective in exploring symbiotic patterns and understanding microbial community structure and assembly dynamics (19–21). Theoretically, microbial communities with more complex associational features are expected to exhibit increased metabolic activity and accelerated growth rates, thereby enhancing overall community performance (22, 23). Microbes associated with seagrasses play a crucial role in seagrass bed ecosystems, contributing to the health of seagrasses and ecosystem functioning (6). Seagrasses evolved from terrestrial flowering plants and transitioned back to marine environments approximately 100 million years ago (24). Similar to terrestrial plants, seagrasses harbor microbial communities in different plant tissues, including the rhizosphere, endosphere, and phyllosphere, forming a holobiont with the plant host (25–27). Extensive research on interactions between terrestrial plants and microbes indicates that the presence of specific microbes significantly impacts plant growth, health, and yield (27–29). Studies suggest that seagrasses can influence their associated microbial communities through nutrient secretion while also benefiting from these communities (25, 30, 31). The intimate association between hosts and their associated microbial communities conceptualizes them as functionally complex units capable of co-adapting to environmental fluctuations (32, 33). However, compared to studies on terrestrial plant-microbe communities, research on different microhabitats of microbial communities in seagrasses is relatively limited. Therefore, in this study, we utilize co-occurrence network analysis to explore co-occurrence patterns of microbial communities in different microhabitats of seagrass beds.

Keystone taxa play a pivotal role in communities, and their removal can result in significant changes to community structure and function (34). It is important to note that the concept of “keystone taxa” has been defined in multiple ways (35). The original concept of keystone species was introduced by Paine in his study of aquatic food web ecology (36). Subsequently, Power et al. (37) provided a broader and more concise definition, describing keystone species as those that have a disproportionately large impact relative to their abundance. Banerjee et al. (38) defined keystone taxa in microbial ecology as highly connected taxa that exert significant influence on the structure and function of the microbiome, regardless of their abundance across spatial and temporal scales. These taxa play a unique and critical role in microbial communities, either individually or as a group, and their removal could lead to substantial disruptions in the structure and functionality of the microbiome. In microbial networks, keystone taxa are determined by their within-module connectivity (Zi) and among-module connectivity (Pi) scores, including module hubs, connectors, and network hubs (39, 40). They are highly associated with high average degree, high closeness centrality, and low betweenness centrality scores (41). Keystone taxa can drive changes in microbial community structure and ecosystem functions (38, 42, 43). For instance, keystone taxa can significantly influence the diversity and functions of ammonia-oxidizing archaea and bacterial communities (44), and keystone taxa can notably impact the multifunctionality of soil in tea plantation ecosystems (45). Therefore, investigating the relationships between keystone taxa and the structure and ecological functions of microbial communities in different microhabitats is crucial for a deeper understanding of microbial communities. In this study, high-throughput sequencing techniques were employed to investigate the microbial communities associated with two seagrass species (*Phyllospadix iwatensis* and *Zostera marina*) in different microhabitats (rhizosphere and phyllosphere) across different periods. Building upon existing research, we formulated the following hypotheses: (i) Do differences in seagrass microhabitats influence the associative characteristics of microbial networks? (ii) Do variations in seagrass microhabitats affect the diversity of keystone groups within microbial co-occurrence networks? If so, might these keystone groups play distinct roles in the structure of microbial communities?

## MATERIALS AND METHODS

### Sample collection

**Fig 1:**
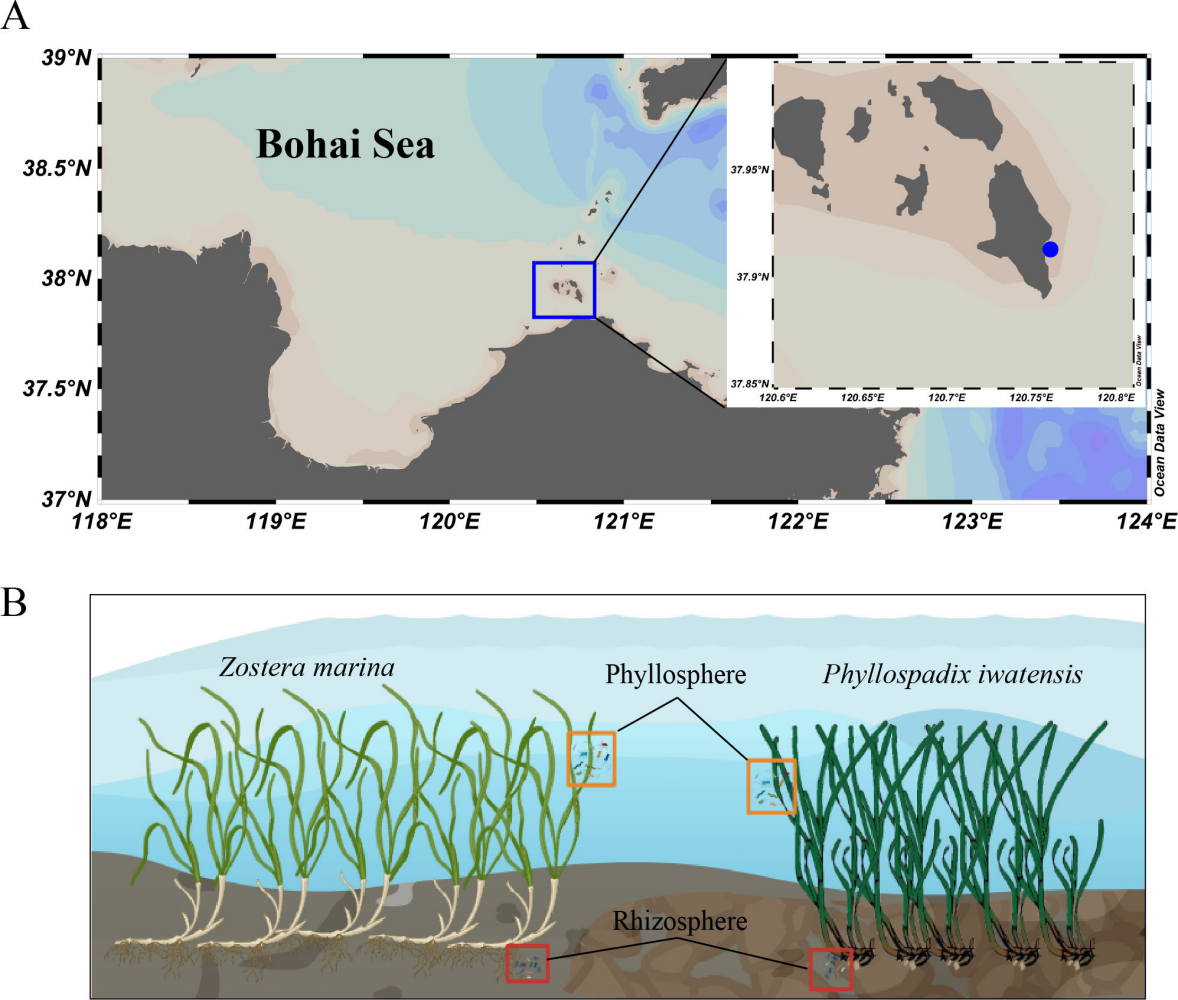
Sample information. (**A**) Sampling location (blue dot indicates the sampling area). (**B**) The distribution patterns of two seagrass species and the microbial sampling regions.

Based on previous investigations into the growth cycles of seagrass beds, we conducted sampling in April and July 2021 in the intertidal zone of Changdao County (Fig. 1), 37.91326°N, 120.75894°E. The sampling periods corresponded to the growth and reproductive stages of two seagrass species (*Zostera marina* and *Phyllospadix iwatensis*). Samples of rhizosphere and phyllosphere were collected during low tide. Rhizosphere and phyllosphere samples from seagrasses in the growth stage were labeled as SR and SP, respectively, while those from seagrasses in the reproductive stage were labeled as FR and FP. The two seagrasses grow separately, and the sampling locations for the two seagrass species were approximately 10 meters apart. For the collection of rhizosphere samples, we manually agitated selected seagrass roots to remove loose sediment and rinsed them with seawater to collect the microorganisms still attached to the rhizosphere (46–48). For phyllosphere samples, sterile disposable forceps were used to randomly select seagrass leaves of the same age class and height, followed by wiping the surface of healthy tissue leaves devoid of algal epiphytes with a sterile swab for sampling (49). Three replicate samples were collected at each sampling point. Each sample was collected in cryogenic tubes, immediately stored in liquid nitrogen on-site, and subsequently transferred to a laboratory freezer at −80℃ for further analysis. Three replicate samples were collected at each sampling point. Seagrass samples were stored in portable refrigerators and subsequently transferred to the laboratory for further analysis.

### DNA extraction and 16S rRNA gene and ITS gene sequencing

Total genomic DNA was extracted from each sample using the FastDNA SPIN Kit (MP Biomedicals, Solon, United States) following the manufacturer’s protocol. The quality and concentration of the DNA extract were assessed using a NanoDrop 2000 UV-vis spectrophotometer (Thermo Fisher Scientific, Wilmington, United States) and confirmed by running an agarose gel electrophoresis with 1% agarose gel. The V3-V4 region of the 16S rRNA genes was amplified using the primer pairs 338F (5′-ACTCCTACGGGAGGCAGCAG-3′) and 806R (5′-GGACTACHVGGGTWTCTAAT-3′) on an ABI GeneAmp 9700 PCR thermocycler (ABI, CA, United States). For the amplification of the fungal ITS1 region, the primer pairs ITS1F (5′-CTTGGTCATTTAGAGGAAGTAA-3′) and ITS2R (5′-GCTGCGTTCTTCATCGATGC-3′) were used with the same PCR thermocycler. PCR products were purified by agarose gel extraction using the AxyPrep DNA Gel Extraction Kit (Axygen Biosciences, Union City, CA, United States) according to the manufacturer’s instructions. The purified DNA was quantified using a Quantus Fluorometer (Promega, United States). The bacterial rRNA genes and fungal ITS genes were subjected to high-throughput sequencing using the Illumina MiSeq PE300 platform (Illumina, San Diego, United States) at Majorbio Bio-Pharm Technology Co., Ltd. (Shanghai, China). Standard protocols were followed during the sequencing process to ensure the accuracy and reliability of the data.

### Bioinformatics and analysis

The raw sequencing reads of the 16S rRNA and ITS genes were processed using fastp version 0.20.0 (50) for demultiplexing and quality filtering. Subsequently, the filtered reads were merged using FLASH version 1.2.7 (51). Operational taxonomic unit (OTU) clustering was conducted using UPARSE version 7.1 (52) at a 97% similarity threshold on the non-redundant sequences, excluding single sequences. Chimeric sequences were effectively removed during the clustering process. The OTU table was manually filtered, i.e., chloroplast sequences in all samples were removed. The taxonomy of each OTU representative sequence was analyzed by RDP Classifier version 2.2 (53) against the 16S rRNA gene database (Silva v138, http://www.arb-silva.de), ITS gene database (Unite v8.0, http://unite.ut.ee/index.php) using confidence threshold of 0.7.

The alpha diversity indices (Shannon index and Chao index) were calculated for each sample in vegan 2.4.3 with R v. 3.5.3. Differences in bacterial community composition, based on the OTU abundance table, were reduced using non-metric multidimensional scaling (NMDS) with Bray-Curtis distance matrix and tested through permutational multivariate analysis of variance (PERMANOVA) (54). The analysis was carried out using R v. 3.5.3 with the vegan package (version 2.4.3) (55). Linear discriminant analysis combined with effect size measure (LEfSe) analysis was used to search for significantly different biomarkers among different microhabitats using Galaxy (http://galaxy.biobakery.org/) (56, 57). Taxa with an LDA score >4 were considered enriched taxa (58). The multigroup comparison strategy uses all-against-all (more strict). Network analysis was conducted based on Spearman’s correlations calculated using the psych package (59) in R (*P* < 0.05; |R| > 0.4) and visualized using Gephi (60). Based on the values of within-module connectivity (Zi) and among-module connectivity (Pi), each node in the network is categorized into distinct topological roles: module hubs (Zi ≥ 2.5, Pi < 0.62), network hubs (Zi ≥ 2.5, Pi ≥ 0.62), connectors (Zi < 2.5, Pi ≥ 0.62), and peripherals (Zi < 2.5, Pi < 0.62) (39). Apart from peripherals, the other three categories are considered potential keystone taxa due to their significant roles in the network topology (61, 62). Calculations of Zi and Pi are typically performed in the R environment. Null-model analysis is employed to assess the contributions of stochastic and deterministic processes to microbial community assembly (7, 63). By utilizing phylogenetic turnover between samples and null models to infer ecological processes and compare the contributions of deterministic and stochastic assembly processes on bacterial communities, a significant phylogenetic signal is essential (64). The correlation between ecological similarity and phylogenetic similarity among species results in closely related taxa exhibiting more similar habitat preferences than distantly related taxa, thus giving rise to the phylogenetic signal. Through the computation of the abundance-weighted average of environmental variables for each OTU, we were able to determine the optimal environmental parameters for each OTU, providing an approximation of the ecological niche values for each OTU. The phylogenetic distances between OTUs were assessed using the “picante,” with the Euclidean distance serving as a measure of the differences in optimal environmental parameters between OTUs. The correlation coefficient between optimal environmental values and phylogenetic distance differences was evaluated using mantel correlograms in the vegan package (65, 66). The nearest taxon index (NTI) is utilized to evaluate the phylogenetic relatedness of taxa within the community, with computations facilitated using the “picante” package (67). βNTI (68) and RC_bray_ (8) values are used to quantify the impacts of deterministic and stochastic processes. In order to quantify the extent to which βMNTD deviates from the expected null model, we utilized a randomization approach by sorting species names and abundances at the tips of the phylogeny and repeating this process 999 times. The difference between the observed βMNTD and the average βMNTD expected from the null model was measured in standard deviation units, known as βNTI (beta nearest-taxon index). A βNTI > 2 or βNTI < −2 signifies a significant deviation of microbial communities from the anticipated phylogenetic turnover, indicating that turnover between communities is primarily determined by deterministic processes. Specifically, a βNTI < −2 suggests that the phylogenetic relationships among OTUs within microbial communities are closer than expected from the null model, with co-occurrence of OTUs driven by homogenizing environmental selection. Conversely, a βNTI > 2 implies that the phylogenetic relationships among OTUs between microbial communities are more distantly related than the null model, and the co-occurrence of OTUs is influenced by heterogenizing environmental selection. The relative contribution of stochastic processes is assessed by comparing the percentage of pairwise community comparisons with |βNTI| < 2, indicating that the observed βMNTD does not significantly deviate from the null model’s βMNTD. The Raup-Crick (RC_bray_) value based on Bray-Curtis is employed to characterize the distance values of observed species composition levels calculated using the Bray-Curtis dissimilarity matrix coefficient and the average value computed based on null models (69). A value of |RC_bray_| > 0.95 is considered indicative of dispersal, encompassing homogenizing dispersal (RC_bray_ < −0.95) and dispersal limitation (RC_bray_ > 0.95); if |βNTI| < 2 and |RC_bray_| < 0.95, then drift (referred to as the “undominated process”) drives the turnover between communities (69). These values are computed using the “pNST” function within the “NST” package. The Wilcoxon rank-sum test is employed to analyze the significance of differences between two sets of data. Multiple tests were corrected for FDR using a two-tailed test.

## RESULTS

### Community diversity and composition in different microhabitats of seagrasses

**Fig 2:**
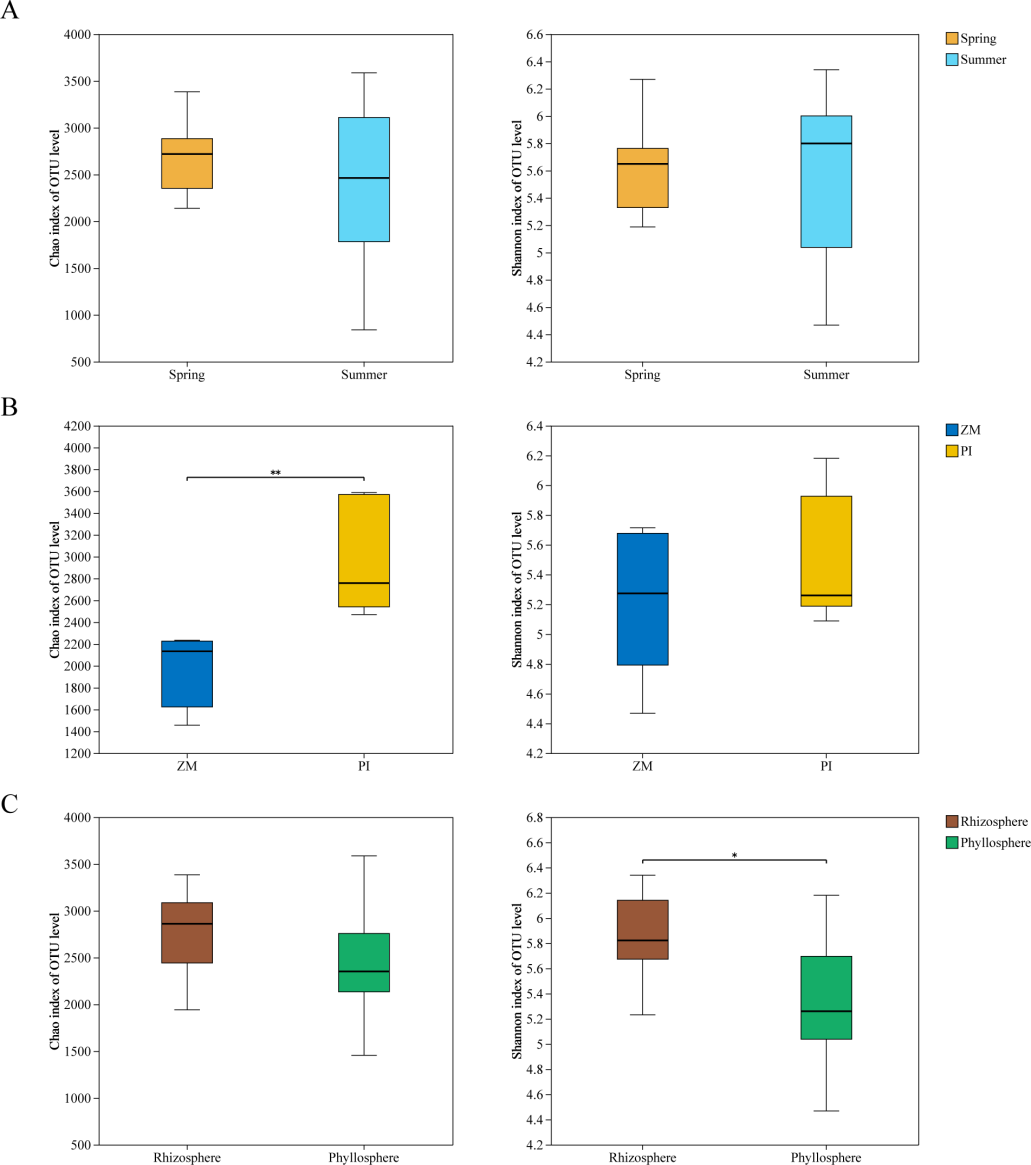
(**A**) Shannon and Chao diversity of bacterial communities in two periods of seagrass. (**B**) Shannon and Chao diversity of bacterial communities in two seagrass species, where ZM is *Zostera marina* and PI is *Phyllospadix iwatensis*. (**C**) Shannon and Chao diversity of the bacterial communities between rhizosphere and phyllosphere. Boxplot showing 12 samples (*n* = 12). Statistical comparisons of the data were conducted using the Wilcoxon rank-sum test. Significance levels were denoted as follows: * *P* < 0.05, ** *P* < 0.01, and *** *P* < 0.001.

**Fig 3:**
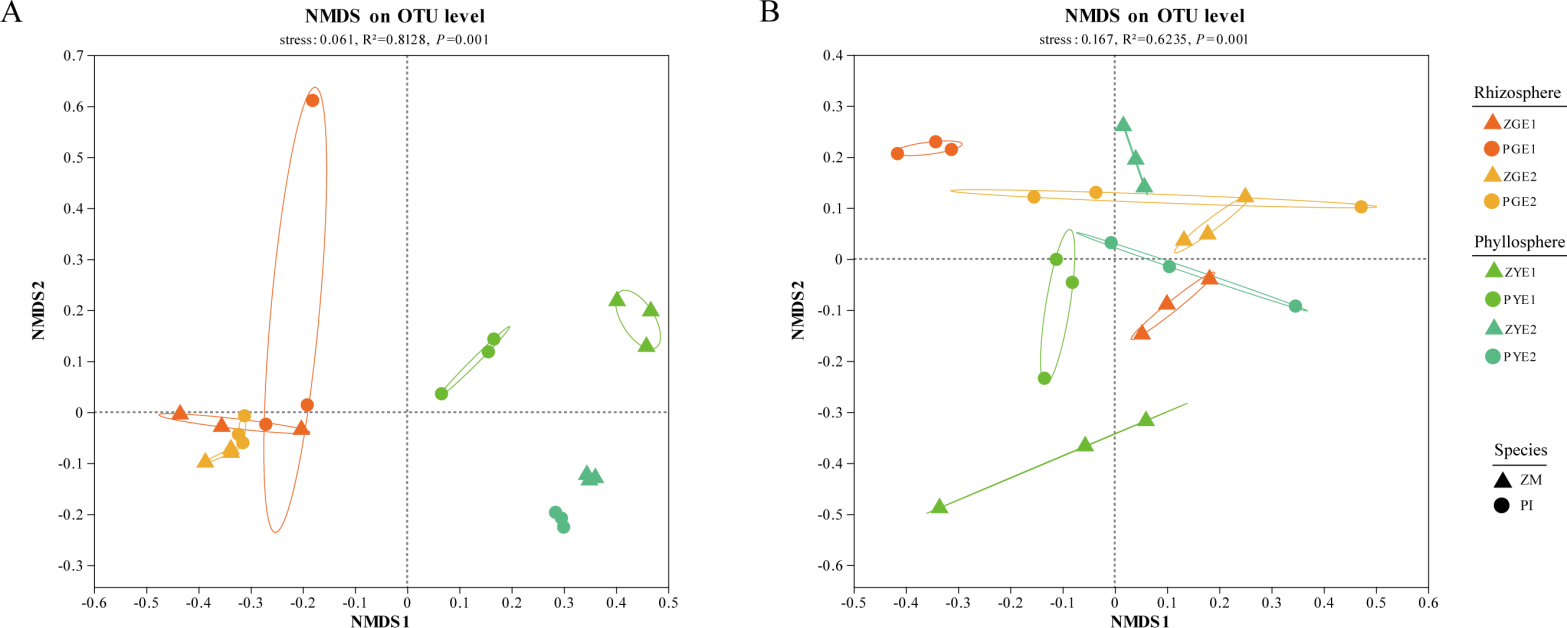
Based on the Bray-Curtis distance metric, NMDS analysis was conducted to examine the bacterial (**A**) and fungal (**B**) communities in the rhizosphere and phyllosphere of two seagrass species (*Zostera marina* and *Phyllospadix iwatensis*). Each group consisted of three samples (*n* = 3) representing the rhizosphere (ZGE1, PGE1, ZGE2, and PGE2) and phyllosphere (ZYE1, PYE1, ZYE2, and PYE2) of the seagrasses. In the NMDS plot, samples that are closer together represent a higher similarity in community composition.

The results indicate that the richness and diversity of seagrass microbial communities during different periods are not significantly different (Fig. 2A; Fig. S1). The bacterial community richness of *Phyllospadix iwatensis* is notably higher than that of *Zostera marina*, while diversity shows no significant difference (Fig. 2B). It is noteworthy that the diversity of seagrass microbial communities in the rhizosphere is higher than that in the phyllosphere (Wilcoxon rank-sum test, *P* < 0.05) (Fig. 2C). NMDS analysis revealed substantial differences in microbial community composition between rhizosphere and phyllosphere samples across different time periods (Fig. 3). PERMANOVA results emphasized the pronounced impact of microhabitat on seagrass microbial community composition relative to species and period (Tables S1 and S2), particularly within the bacterial community.

**Fig 4:**
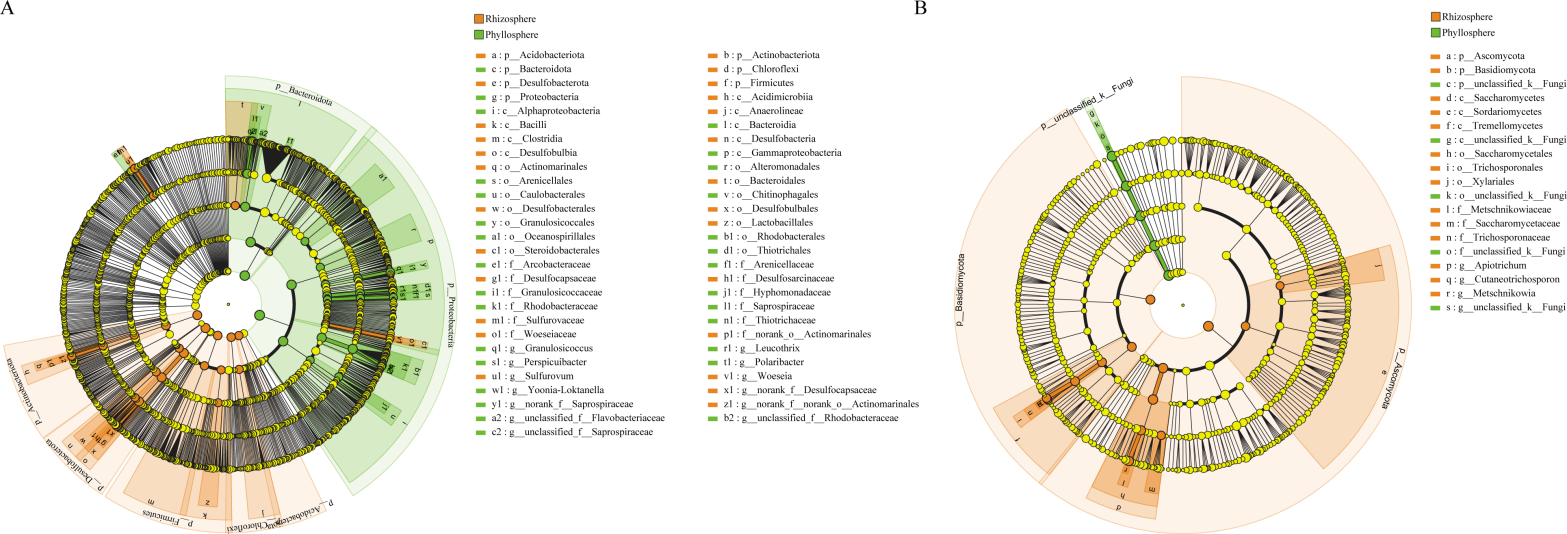
LEfSe analysis results for the bacterial (**A**) and fungal (**B**) communities in the samples collected from the seagrass rhizosphere and phyllosphere.

LEfSe analysis unveiled significant enrichments of Desulfobacterota, Actinobacteriota, Chloroflexi, Firmicutes, and Acidobacteriota in the rhizosphere bacterial community (Fig. 4A), Proteobacteria and Bacteroidota were notably enriched in the phyllosphere bacterial community. Furthermore, Ascomycota and Basidiomycota in the rhizosphere fungal community (Fig. 4B), while unclassified_k__Fungi exhibited significant enrichment in the phyllosphere fungal community.

### Interaction and co-occurrence networks of the bacterial and fungal communities

**Fig 5:**
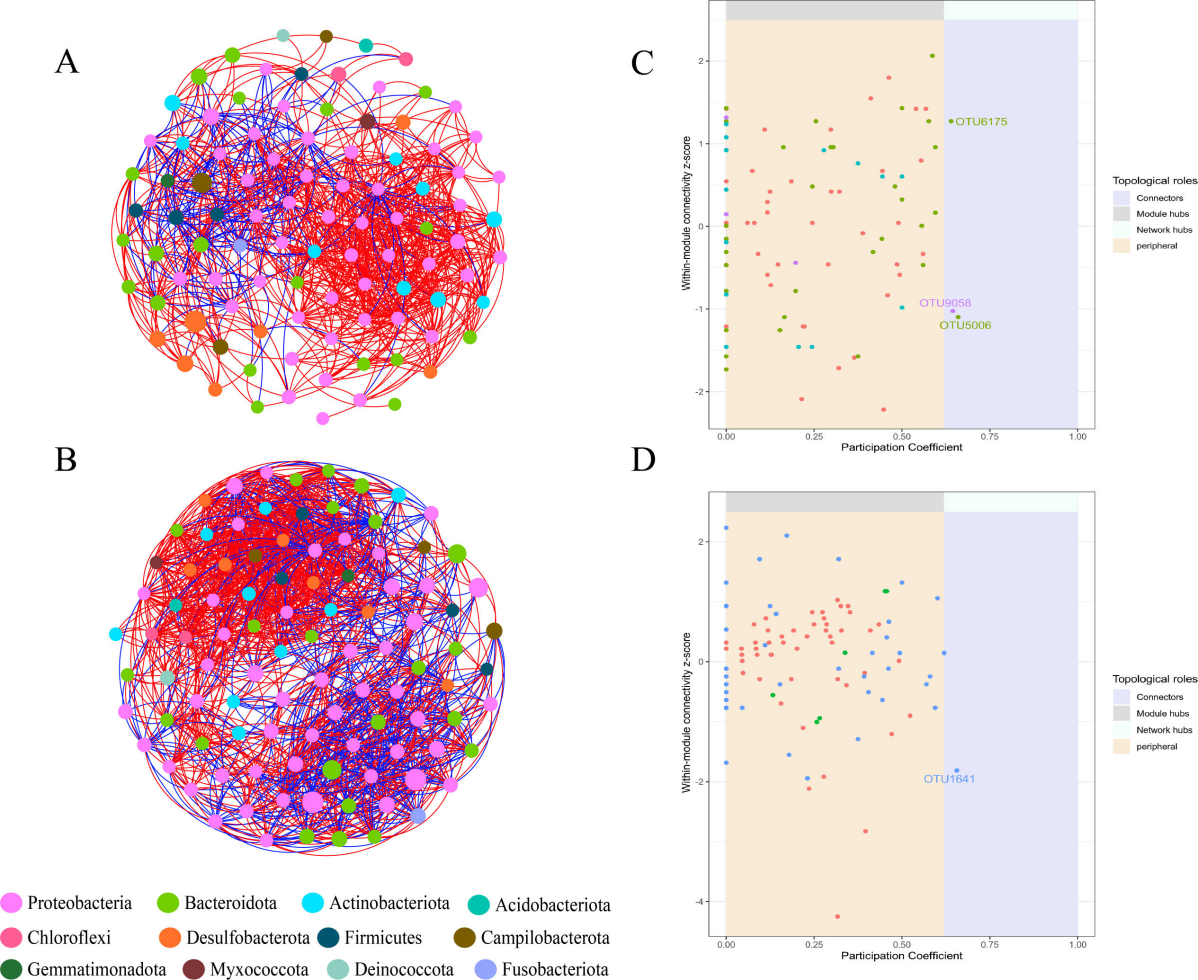
Co-occurrence network and keystone taxa of bacterial communities at the OTU levels in rhizosphere samples (**A** and **C**) and phyllosphere samples (**B** and **D**). The top 100 OTUs for relative abundance were selected for each sample. Nodes in the network are color-coded for different bacterial phyla, while lines connecting nodes represent correlations between OTUs. Positive correlations are indicated by red lines and negative ones by blue lines. Specifically, module hubs were defined as taxa with Zi ≥ 2.5 and Pi < 0.62, connectors as taxa with Zi < 2.5 and Pi ≥ 0.62, and network hubs as taxa with Zi ≥ 2.5 and Pi ≥ 0.62.

In addition, we constructed microbial co-occurrence networks in the rhizosphere and phyllosphere based on 100 OTUs with high relative abundance (Fig. 5; Fig. S2). Lower edge number and network density compared to the rhizosphere microbial community. The bacterial co-occurrence networks co-occurrence networks had greater positive than negative proportions in the rhizosphere and phyllosphere, and the fungal co-occurrence network showed the same results (Table S3). The bacterial community network in the phyllosphere surpasses that of the rhizosphere in terms of nodes (100 vs 97), edges (1,460 vs 895), and modularity (0.403 vs 0.354). An increase in the number of edges may indicate more frequent interactions among phyllosphere bacterial communities, potentially driven by the availability of new resources in the environment, which fosters greater cooperation or competition among microorganisms. The observed increase in modularity suggests that the phyllosphere bacterial communities are forming tighter functional units in response to environmental changes. Additionally, the phyllosphere bacterial network exhibits a higher average clustering coefficient (0.663 vs 0.596), indicating more complex interactions within the microbial communities. Biologically, this suggests that the structure of the phyllosphere bacterial community is relatively cohesive. Subsequently, based on topological roles, we further investigated the variations of keystone taxa within the microbial communities of seagrass phyllospheres and rhizospheres (Fig. 5). In the bacterial co-occurrence network of the rhizosphere, three keystone taxa were identified (Table S4): *Pseudoruegeria*, *Ilumatobacter*, and *norank_norank_Subgroup_17*. In the bacterial co-occurrence network of the phyllosphere, *Algibacter* emerged as a keystone taxon. Within the fungal co-occurrence networks of the phyllosphere and rhizosphere, one and six keystone taxa were identified, respectively (Fig. S2).

### Major processes of microbial community assembly in different microhabitats

**Fig 6:**
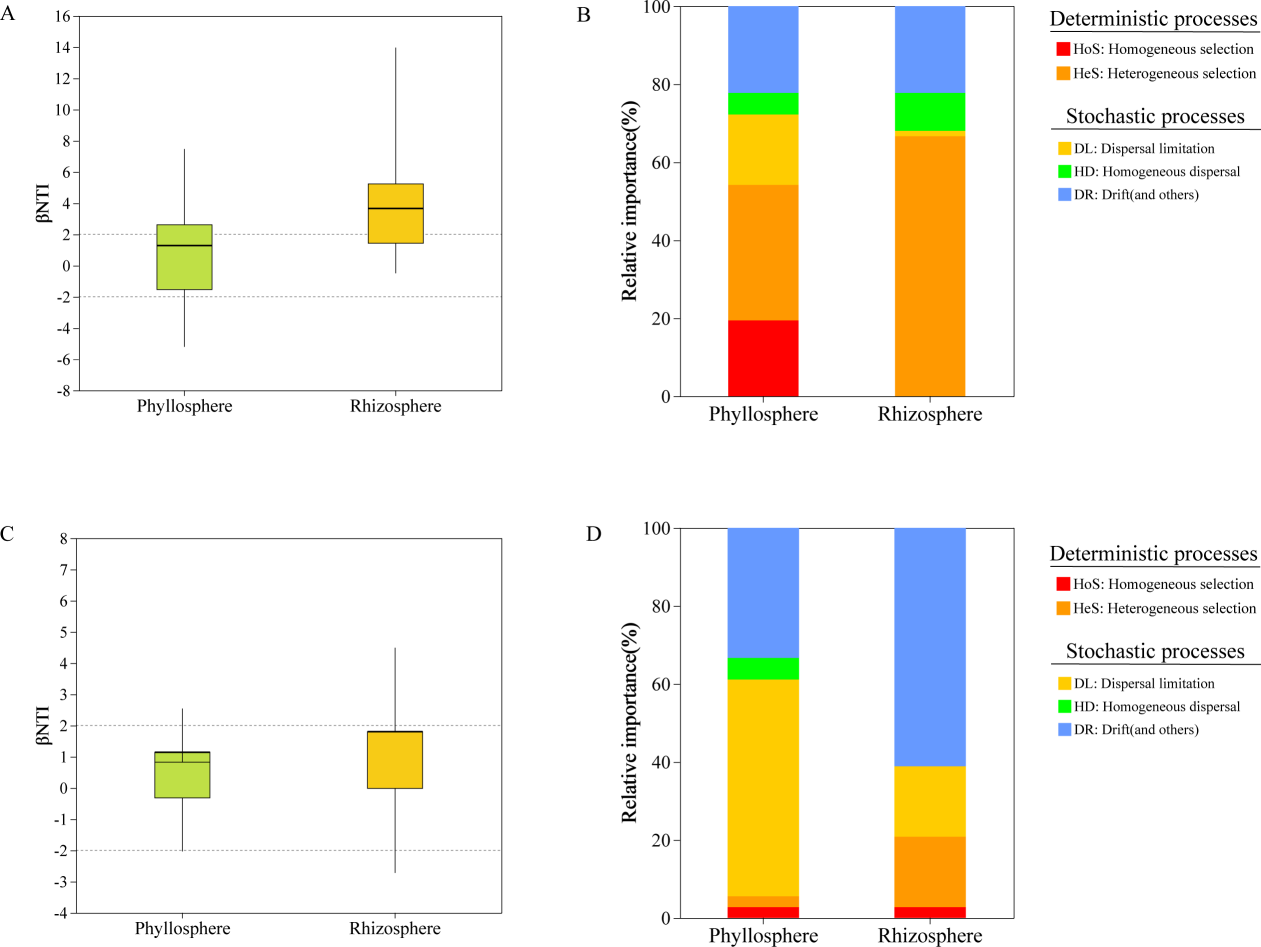
Evaluation of the assembly mechanisms of bacterial communities and fungal communities in the phyllosphere and rhizosphere by using null model analysis. The contributions of deterministic processes (|βNTI| ≥ 2) and random processes (|βNTI| < 2) to the assembly of bacterial communities (**A**) and fungal communities (**C**) assembly in the phyllosphere and rhizosphere. The relative contributions of different ecological processes drive the assembly of bacterial communities (**B**) and fungal communities (**D**) in the phyllosphere and rhizosphere.

By utilizing null models, we investigated the assembly processes of microbial communities in the rhizosphere and phyllosphere of seagrasses. Phylogenetic signals indicate the presence of significant phylogenetic relationships within short phylogenetic distances in both the rhizosphere and phyllosphere microbial communities of seagrass beds (Fig. S3). The results showed that in the rhizosphere, deterministic processes (|βNTI| > 2) predominantly dominated the assembly of bacterial communities (Fig. 6A), while stochastic processes dominated the phyllosphere. Regarding different microhabitats of seagrasses, the proportion of heterogeneous selection in the rhizosphere is significantly higher than that in the phyllosphere (Fig. 6B). Subsequently, we delved further into the assembly of microbial communities in different microhabitats of seagrasses across different periods. We observed that as the reproductive period approaches, the proportion of heterogeneous selection in the assembly of bacterial communities in both the phyllosphere and rhizosphere gradually increases (Fig. S4B). In contrast, the assembly of fungal communities in both the rhizosphere and phyllosphere is primarily driven by stochastic processes (Fig. 6C). Moreover, in the phyllosphere, dispersal limitation accounts for a higher proportion of stochastic processes, whereas in the rhizosphere, drift and other factors occupy a larger proportion within stochastic processes (Fig. 6D). Additionally, following the reproductive period of seagrasses, the proportion of deterministic processes in the assembly of fungal communities in both the phyllosphere and rhizosphere decrease, while the proportion of dispersal limitation increases (Fig. S4D).

## DISCUSSION

The research findings indicate that the microbial diversity in the rhizosphere exceeds that of the phyllosphere, consistent with previous studies on seagrass microbial communities (70). Additionally, PERMANOVA analysis revealed that microhabitat was the most dominant effect on seagrass microbial community composition relative to periods and species. These findings align with previous studies emphasizing the microbial habitat specificity in seagrass (14). Furthermore, co-occurrence network analysis reveals significant influences of different microhabitats on the microbial community structure of seagrasses. The bacterial community network in the phyllosphere surpasses that of the rhizosphere in terms of nodes, edges, and modularity. Previous studies suggest that topologically-based modules in microbial networks can be considered functional units of microbial communities, where these modules represent potential ecological niches of the microbial community (71, 72). This increase in modularity may reflect the presence of more dense functional associations and/or ecological niche partitioning within the bacterial community of seagrass phyllospheres. Additionally, the phyllosphere bacterial network exhibits a higher average clustering coefficient. These results suggest that any two bacteria within the bacterial community of the phyllosphere can be linked by a small number of neighboring species, demonstrating “small-world” characteristics. In a small-world system, highly connected nodes are few, allowing for rapid transmission of perturbations throughout the entire community (73–75). This indicates that interactions among bacterial communities in the phyllosphere are more complex compared to the rhizosphere, potentially leading to intricate dynamics within the microbial community.

Seagrass leaves and roots release nutrients to attract bacteria to colonize the plant surfaces (76, 77). Variations in the released nutrients may partially influence the dispersion of bacterial communities, leading to selective enrichment of microbial populations in different microhabitats (78–80). Research indicates that bacteria isolated from *Zostera marina* roots exhibit chemotaxis toward amino acids exuded by eelgrass roots (81). We found the enrichment of Desulfobacterota and Actinobacteria was observed in the rhizosphere, while Proteobacteria and Bacteroidota were enriched in the phyllosphere. The variation in different bacterial groups between the rhizosphere and phyllosphere represents their microhabitat preferences, aligning with similar studies (51, 82, 83). Further studies found that, a significant enrichment of *Sulfurovum* and *g__norank_f__Desulfocapsaceae* in the rhizosphere of seagrasses (Fig. 4A), which are associated with sulfur-oxidizing bacteria and sulfate-reducing bacteria (84, 85). Members of the genus *Sulfurovum* are considered to be important players that couple sulfide-oxidation and denitrification/sulfide-oxidation and carbon fixation processes in marine environments and have been frequently observed in the rhizospheres of marine plants and seagrass (48, 86). The interactions between sulfide-oxidizing bacteria and seagrasses are putatively mutualistic and may be critical to seagrass growth in sulfide-rich sediments (87, 88). Previous studies have demonstrated that keystone species have a significant impact on microbial community structure and ecosystem function (35, 89). Different keystone taxa were observed in the rhizosphere and phyllosphere of seagrass in this study. Changes in microhabitats may alter the keystone taxa within specific environments, with no overlapping species acting as keystones across multiple networks. This suggests that keystone taxa as central hubs in networks may be specific to particular microhabitats. Some studies have also noted the existence of specific OTUs only during certain seasons or time periods (20, 90). Therefore, we speculate that the reasons why microbial species become keystone taxa are associated with specific environmental conditions rather than environmental changes. Keystone taxa within the rhizosphere bacterial communities were found to potentially participate in sulfur cycling. Despite limited research, *Ilumatobacter* has gained recognition for its presence in coastal environments (91, 92). Zhang et al. (93) observed a significant contribution of Ilumatobacter to sulfate reduction in sulfur cycling processes. Liu et al. (94) linked Pseudoruegeria to sulfide oxidation (*sqr*). In the phyllosphere bacterial community, *Algibacter_lectus* has been identified as a keystone taxon. Research has shown that *Algibacter_lectus* possesses the ability to degrade complex organic compounds such as polysaccharides, cellulose, and algal-related polymers, particularly the degradation of polysaccharides secreted by marine algae (e.g., alginates, carrageenans) (95). We hypothesize that the degradation ability of *Algibacter_lectus* could lead to changes in the way carbon sources are utilized within the seagrass leaf-associated microhabitat. Microorganisms capable of using their degradation products as carbon sources may have gained a competitive advantage, increasing their survival and reproduction opportunities. As a result, their proportion within the microbial community might increase, ultimately altering the overall community structure. This observation aligns with our finding that seagrass phyllosphere microbial communities exhibit lower diversity but more complex interactions. However, further methods such as metagenomics and metatranscriptomics are needed to validate the identification and functional roles of potential key taxa within networks (96, 97).

Microbial community assembly is likely influenced by multiple ecological processes simultaneously, with varying contributions of different ecological processes in different community compositions (98). In order to quantitatively assess the contributions of different ecological processes to community assembly more scientifically, we inferred the ecological processes of microbial communities in different microhabitats of seagrasses during different periods using null models (69). Notably, heterogeneous selection occupies different proportions of the assembly of microbial communities in different microhabitats. We observed a higher proportion of deterministic processes (|βNTI| > 2) in the assembly of rhizosphere bacterial communities, whereas the assembly of phyllosphere bacterial communities was predominantly influenced by stochastic processes. This finding is consistent with the conclusions of Kardish and Stachowicz (16). Many studies assume that community plots sampled at fine spatial scales are environmentally uniform, overlooking the impact of abiotic micro-variations on the observed community and its intricate assembly within the plot (99). However, research has demonstrated that even at such fine scales, environmental factors such as soil properties can exhibit significant heterogeneity, leading to a patchy distribution of uniform “micro-sites,” which in turn influences plant communities (99, 100). This is also consistent with our results. Furthermore, we observed that the impact of deterministic processes on the rhizosphere bacterial community increased from 38.9% in the SR to 77.8% in the FR, primarily driven by heterogeneous selection. This suggests that in the FR, selective pressure intensifies, reducing the role of stochastic processes in bacterial community assembly. We hypothesize that during this period, seagrasses may recruit specific microbes by secreting particular compounds, enhancing selective pressures, and thereby increasing the influence of deterministic processes. During the transition to the reproductive period, seagrasses release phenolic substances and amino acids to recruit specific bacteria (101). In contrast, stochastic processes play a more significant role in the aggregation of fungal communities. Additionally, when there are temporal changes, the response of seagrass bacterial communities appears to be more pronounced compared to fungal communities. This is mainly because most bacteria are r-strategists, with their growth and development highly regulated by nutritional conditions (102). In comparison to bacteria, the assembly process of fungal communities is less influenced by microhabitat factors, which might be a result of k-selection within the fungal community. In contrast to r-strategists, k-strategists efficiently utilize available resources, even when resources are not limited (102, 103). We also consider that changes in ecological processes may be influenced by the reproductive period or direct responses to seasonal environmental changes. Therefore, further experiments are needed to validate these hypotheses.

### Conclusion

This study analyzed the microbial community structures of two seagrass species in different microhabitats at different periods. The results indicate that, compared to variations in seagrass species and periods, the microhabitats of seagrasses have a more significant impact on the composition and structure of the microbial communities. Differences in seagrass microhabitats influenced community assembly, with rhizosphere microbial communities being more influenced by deterministic processes (heterogeneous selection) compared to the phyllosphere. Although the diversity of phyllosphere microbial communities is lower than that of the rhizosphere, their co-occurrence networks exhibit more complex interactions. Furthermore, keystone taxa differ significantly in different seagrass microhabitats.

## Data Availability

The raw sequencing reads were deposited in the NCBI Sequence Read Archive (SRA) database under accession numbers PRJNA1194325 and PRJNA805355.

## SUPPLEMENTAL MATERIAL

Fig. S1 aem.02437-24-s0001.pdf Shannon and Chao diversity of fungal communities in two periods of seagrass, in two seagrass species, and between rhizosphere and phyllosphere.

Fig. S2 aem.02437-24-s0002.pdf Co-occurrence network and keystone taxa of fungal communities at the OTU levels in rhizosphere and phyllosphere samples.

Fig. S3 aem.02437-24-s0003.tif Mantel correlograms between the pairwise of OTU niche distances and phylogenetic distances with 999 permutations (Spearman correlation) in bacterial communities and fungal communities.

Fig. S4 aem.02437-24-s0004.tif Evaluation of the assembly mechanisms of bacterial communities and fungal communities in the SR, FR, SP, and FP by using null model analysis.

Supplemental legends aem.02437-24-s0005.docx Legends for Fig. S1 to S4 and Tables S1 to S4.

Table S1 aem.02437-24-s0006.docx Analysis of different time periods, microhabitats, and seagrass species explaining bacterial community structure (PERMANOVA based on Bray-Curtis distance).

Table S2 aem.02437-24-s0007.docx Analysis of different time periods, microhabitats, and seagrass species explaining fungal community structure (PERMANOVA based on Bray-Curtis distance).

Table S3 aem.02437-24-s0008.docx Properties of co-occurrence networks of bacterial and fungal communities in the rhizosphere and phyllosphere.

Table S4 aem.02437-24-s0009.docx Keystone nodes and their taxonomic information in rhizosphere and phyllosphere.

ASM does not own the copyrights to Supplemental Material that may be linked to, or accessed through, an article. The authors have granted ASM a non-exclusive, world-wide license to publish the Supplemental Material files. Please contact the corresponding author directly for reuse.

## References

[1] Zhou J, Ning D. 2017. Stochastic community assembly: does it matter in microbial ecology? Microbiol Mol Biol Rev 81:e00002-17. doi:10.1128/MMBR.00002-17 29021219 PMC5706748

[2] Wang X, Li Y, Yan Z, Hao Y, Kang E, Zhang X, Li M, Zhang K, Yan L, Yang A, Niu Y, Kang X. 2022. The divergent vertical pattern and assembly of soil bacterial and fungal communities in response to short-term warming in an alpine peatland. Front Plant Sci 13:986034. doi:10.3389/fpls.2022.986034 36160969 PMC9493461

[3] Cao J, Zhang Y, Dai G, Cui K, Wu X, Qin F, Xu J, Dong F, Pan X, Zheng Y. 2023. The long-acting herbicide mesosulfuron-methyl inhibits soil microbial community assembly mediating nitrogen cycling. J Hazard Mater 443:130293. doi:10.1016/j.jhazmat.2022.130293 36444049

[4] Tanunchai B, Ji L, Schroeter SA, Wahdan SFM, Thongsuk K, Hilke I, Gleixner G, Buscot F, Schulze ED, Noll M, Purahong W. 2023. Tree mycorrhizal type regulates leaf and needle microbial communities, affects microbial assembly and co-occurrence network patterns, and influences litter decomposition rates in temperate forest. Front Plant Sci 14:1239600. doi:10.3389/fpls.2023.1239600 38094000 PMC10716483

[5] Loke LHL, Chisholm RA. 2023. Unveiling the transition from niche to dispersal assembly in ecology. Nature 618:537–542. doi:10.1038/s41586-023-06161-x 37286612 PMC10266978

[6] Niu X, Ren W, Xu C, Wang R, Zhang J, Wang H. 2024. Taxonomic and functional β-diversity patterns reveal stochastic assembly rules in microbial communities of seagrass beds. Front Plant Sci 15:1367773. doi:10.3389/fpls.2024.1367773 38481397 PMC10932972

[7] Ning D, Deng Y, Tiedje JM, Zhou J. 2019. A general framework for quantitatively assessing ecological stochasticity. Proc Natl Acad Sci U S A 116:16892–16898. doi:10.1073/pnas.1904623116 31391302 PMC6708315

[8] Stegen JC, Lin X, Fredrickson JK, Chen X, Kennedy DW, Murray CJ, Rockhold ML, Konopka A. 2013. Quantifying community assembly processes and identifying features that impose them. ISME J 7:2069–2079. doi:10.1038/ismej.2013.93 23739053 PMC3806266

[9] Vellend M. 2010. Conceptual synthesis in community ecology. Q Rev Biol 85:183–206. doi:10.1086/652373 20565040

[10] Yarnall AH, Byers JE, Yeager LA, Fodrie FJ. 2022. Comparing edge and fragmentation effects within seagrass communities: a meta-analysis. Ecology 103:e3603. doi:10.1002/ecy.3603 34897663

[11] Han Q, Qiu C, Zeng W, Chen S, Zhao M, Shi Y, Zhang X. 2023. Sediment carbon sequestration and driving factors in seagrass beds from Hainan island and the Xisha islands. Processes (Basel) 11:456. doi:10.3390/pr11020456

[12] Santos RO, Lirman D, Pittman SJ. 2016. Long‐term spatial dynamics in vegetated seascapes: fragmentation and habitat loss in a human‐impacted subtropical lagoon. Mar Ecol 37:200–214. doi:10.1111/maec.12259

[13] Du J, Hu W, Nagelkerken I, Sangsawang L, Loh KH, Ooi J-S, Liao J, Zheng X, Qiu S, Chen B. 2020. Seagrass meadows provide multiple benefits to adjacent coral reefs through various microhabitat functions. Ecosyst Health Sustain 6:1812433. doi:10.1080/20964129.2020.1812433

[14] Banister RB, Schwarz MT, Fine M, Ritchie KB, Muller EM. 2022. Instability and stasis among the microbiome of seagrass leaves, roots and rhizomes, and nearby sediments within a natural pH gradient. Microb Ecol 84:703–716. doi:10.1007/s00248-021-01867-9 34596709 PMC9622545

[15] Walker LD, Gribben PE, Glasby TM, Marzinelli EM, Varkey DR, Dafforn KA. 2024. Above and below-ground bacterial communities shift in seagrass beds with warmer temperatures. Front Mar Sci 11. doi:10.3389/fmars.2024.1374946

[16] Kardish MR, Stachowicz JJ. 2023. Local environment drives rapid shifts in composition and phylogenetic clustering of seagrass microbiomes. Sci Rep 13:3673. doi:10.1038/s41598-023-30194-x 36871071 PMC9985655

[17] Fahimipour AK, Kardish MR, Lang JM, Green JL, Eisen JA, Stachowicz JJ. 2017. Global-scale structure of the eelgrass microbiome. Appl Environ Microbiol 83:e03391-16. doi:10.1128/AEM.03391-16 28411219 PMC5452814

[18] Faust K, Raes J. 2012. Microbial interactions: from networks to models. Nat Rev Microbiol 10:538–550. doi:10.1038/nrmicro2832 22796884

[19] Zheng X, Wang M, Liu X, Xu S, Ma S, Wang J, Wang C, Zhan A, Yu P, Wang D, He Y, Jiang C, Zhuang X. 2024. Revealing assembly mechanisms of algal communities in aquatic microniches: shifts in diversity patterns, microbial interactions and stability along nutrient gradients. Environ Res 262:119798. doi:10.1016/j.envres.2024.119798 39151556

[20] Liu S, Yu H, Yu Y, Huang J, Zhou Z, Zeng J, Chen P, Xiao F, He Z, Yan Q. 2022. Ecological stability of microbial communities in Lake Donghu regulated by keystone taxa. Ecol Indic 136:108695. doi:10.1016/j.ecolind.2022.108695

[21] Xing W, Gai X, Ju F, Chen G. 2023. Microbial communities in tree root-compartment niches under Cd and Zn pollution: structure, assembly process and co-occurrence relationship. Sci Total Environ 860:160273. doi:10.1016/j.scitotenv.2022.160273 36460109

[22] Yu Y, Guo Q, Zhang S, Guan Y, Jiang N, Zhang Y, Mao R, Bai K, Buriyev S, Samatov N, Zhang X, Yang W. 2024. Maize residue retention shapes soil microbial communities and co-occurrence networks upon freeze-thawing cycles. PeerJ 12:e17543. doi:10.7717/peerj.17543 38887621 PMC11182024

[23] Chen J, Li Z, Xu D, Xiao Q, Liu H, Li X, Chao L, Qu H, Zheng Y, Liu X, Wang P, Bao Y. 2023. Patterns and drivers of microbiome in different rock surface soil under the volcanic extreme environment. Imeta 2:e122. doi:10.1002/imt2.122 38867933 PMC10989942

[24] Mohr W, Lehnen N, Ahmerkamp S, Marchant HK, Graf JS, Tschitschko B, Yilmaz P, Littmann S, Gruber-Vodicka H, Leisch N, Weber M, Lott C, Schubert CJ, Milucka J, Kuypers MMM. 2021. Terrestrial-type nitrogen-fixing symbiosis between seagrass and a marine bacterium. Nature 600:105–109. doi:10.1038/s41586-021-04063-4 34732889 PMC8636270

[25] Ugarelli K, Chakrabarti S, Laas P, Stingl U. 2017. The seagrass holobiont and its microbiome. Microorganisms 5:81. doi:10.3390/microorganisms5040081 29244764 PMC5748590

[26] Vogel MA, Mason OU, Miller TE. 2021. Composition of seagrass phyllosphere microbial communities suggests rapid environmental regulation of community structure. FEMS Microbiol Ecol 97:fiab013. doi:10.1093/femsec/fiab013 33493257

[27] Tarquinio F, Hyndes GA, Laverock B, Koenders A, Säwström C. 2019. The seagrass holobiont: understanding seagrass-bacteria interactions and their role in seagrass ecosystem functioning. FEMS Microbiol Lett 366:fnz057. doi:10.1093/femsle/fnz057 30883643

[28] Compant S, Duffy B, Nowak J, Clément C, Barka EA. 2005. Use of plant growth-promoting bacteria for biocontrol of plant diseases: principles, mechanisms of action, and future prospects. Appl Environ Microbiol 71:4951–4959. doi:10.1128/AEM.71.9.4951-4959.2005 16151072 PMC1214602

[29] Hayat R, Ali S, Amara U, Khalid R, Ahmed I. 2010. Soil beneficial bacteria and their role in plant growth promotion: a review. Ann Microbiol 60:579–598. doi:10.1007/s13213-010-0117-1

[30] O’Donohue MJ, Moriarty DJ, Rae IC. 1991. Nitrogen fixation in sediments and the rhizosphere of the seagrass Zostera capricorni. Microb Ecol 22:53–64. doi:10.1007/BF02540212 24194325

[31] Wang X, Chen RF, Cable JE, Cherrier J. 2014. Leaching and microbial degradation of dissolved organic matter from salt marsh plants and seagrasses. Aquat Sci 76:595–609. doi:10.1007/s00027-014-0357-4

[32] Conte C, Rotini A, Manfra L, D’Andrea MM, Winters G, Migliore L. 2021. The seagrass holobiont: what we know and what we still need to disclose for its possible use as an ecological indicator. Water (Basel) 13:406. doi:10.3390/w13040406

[33] Zilber-Rosenberg I, Rosenberg E. 2008. Role of microorganisms in the evolution of animals and plants: the hologenome theory of evolution. FEMS Microbiol Rev 32:723–735. doi:10.1111/j.1574-6976.2008.00123.x 18549407

[34] Mouquet N, Gravel D, Massol F, Calcagno V. 2013. Extending the concept of keystone species to communities and ecosystems. Ecol Lett 16:1–8. doi:10.1111/ele.12014 23062191

[35] Cottee-Jones HEW, Whittaker RJ. 2012. Perspective: the keystone species concept: a critical appraisal. Front Biogeogr 4. doi:10.21425/F54312533

[36] Paine RT. 1966. Food web complexity and species diversity. Am Nat 100:65–75. doi:10.1086/282400

[37] Power ME, Tilman D, Estes JA, Menge BA, Bond WJ, Mills LS, Daily G, Castilla JC, Lubchenco J, Paine RT. 1996. Challenges in the quest for keystones: identifying keystone species is difficult—but essential to understanding how loss of species will affect ecosystems. BioScience 46:609–620. doi:10.2307/1312990

[38] Banerjee S, Schlaeppi K, van der Heijden MGA. 2018. Keystone taxa as drivers of microbiome structure and functioning. Nat Rev Microbiol 16:567–576. doi:10.1038/s41579-018-0024-1 29789680

[39] Olesen JM, Bascompte J, Dupont YL, Jordano P. 2007. The modularity of pollination networks. Proc Natl Acad Sci USA 104:19891–19896. doi:10.1073/pnas.0706375104 18056808 PMC2148393

[40] Zhou J, Deng Y, Luo F, He Z, Yang Y. 2011. Phylogenetic molecular ecological network of soil microbial communities in response to elevated CO_2_. MBio 2:e00122-11. doi:10.1128/mBio.00122-11 21791581 PMC3143843

[41] Berry D, Widder S. 2014. Deciphering microbial interactions and detecting keystone species with co-occurrence networks. Front Microbiol 5:219. doi:10.3389/fmicb.2014.00219 24904535 PMC4033041

[42] Fan K, Delgado-Baquerizo M, Guo X, Wang D, Wu Y, Zhu M, Yu W, Yao H, Zhu Y-G, Chu H. 2019. Suppressed N fixation and diazotrophs after four decades of fertilization. Microbiome 7:143. doi:10.1186/s40168-019-0757-8 31672173 PMC6824023

[43] Herren CM, McMahon KD. 2018. Keystone taxa predict compositional change in microbial communities. Environ Microbiol 20:2207–2217. doi:10.1111/1462-2920.14257 29708645

[44] Yang F, Chen Q, Zhang Q, Long C, Jia W, Cheng X. 2021. Keystone species affect the relationship between soil microbial diversity and ecosystem function under land use change in subtropical China. Funct Ecol 35:1159–1170. doi:10.1111/1365-2435.13769

[45] Han Z, Xu P, Li Z, Lin H, Zhu C, Wang J, Zou J. 2022. Microbial diversity and the abundance of keystone species drive the response of soil multifunctionality to organic substitution and biochar amendment in a tea plantation. GCB Bioenergy 14:481–495. doi:10.1111/gcbb.12926

[46] Lundberg DS, Lebeis SL, Paredes SH, Yourstone S, Gehring J, Malfatti S, Tremblay J, Engelbrektson A, Kunin V, Del Rio TG, Edgar RC, Eickhorst T, Ley RE, Hugenholtz P, Tringe SG, Dangl JL. 2012. Defining the core Arabidopsis thaliana root microbiome. Nature 488:86–90. doi:10.1038/nature11237 22859206 PMC4074413

[47] Costa R, Götz M, Mrotzek N, Lottmann J, Berg G, Smalla K. 2006. Effects of site and plant species on rhizosphere community structure as revealed by molecular analysis of microbial guilds. FEMS Microbiol Ecol 56:236–249. doi:10.1111/j.1574-6941.2005.00026.x 16629753

[48] Zhang X, Zhao C, Yu S, Jiang Z, Liu S, Wu Y, Huang X. 2020. Rhizosphere microbial community structure is selected by habitat but not plant species in two tropical seagrass beds. Front Microbiol 11:161. doi:10.3389/fmicb.2020.00161 32194512 PMC7065525

[49] Vogel MA, Mason OU, Miller TE. 2020. Host and environmental determinants of microbial community structure in the marine phyllosphere. PLoS One 15:e0235441. doi:10.1371/journal.pone.0235441 32614866 PMC7332025

[50] Chen S, Zhou Y, Chen Y, Gu J. 2018. fastp: an ultra-fast all-in-one FASTQ preprocessor. Bioinformatics 34:i884–i890. doi:10.1093/bioinformatics/bty560 30423086 PMC6129281

[51] Markovski M, Najdek M, Herndl GJ, Korlević M. 2022. Compositional stability of sediment microbial communities during a seagrass meadow decline. Front Mar Sci 9:966070. doi:10.3389/fmars.2022.966070

[52] Edgar RC. 2013. UPARSE: highly accurate OTU sequences from microbial amplicon reads. Nat Methods 10:996–998. doi:10.1038/nmeth.2604 23955772

[53] Wang Q, Garrity GM, Tiedje JM, Cole JR. 2007. Naive Bayesian classifier for rapid assignment of rRNA sequences into the new bacterial taxonomy. Appl Environ Microbiol 73:5261–5267. doi:10.1128/AEM.00062-07 17586664 PMC1950982

[54] Oksanen J. 2009. Vegan: community ecology package. R package version 1. 15-4. http://CRAN R-project org/package= vegan.

[55] Chen J, Zang Y, Yang Z, Qu T, Sun T, Liang S, Zhu M, Wang Y, Tang X. 2022. Composition and functional diversity of epiphytic bacterial and fungal communities on marine macrophytes in an intertidal zone. Front Microbiol 13:839465. doi:10.3389/fmicb.2022.839465 35369473 PMC8972133

[56] Segata N, Izard J, Waldron L, Gevers D, Miropolsky L, Garrett WS, Huttenhower C. 2011. Metagenomic biomarker discovery and explanation. Genome Biol 12:1–18. doi:10.1186/gb-2011-12-6-r60 PMC3218848 21702898

[57] Louca S, Parfrey LW, Doebeli M. 2016. Decoupling function and taxonomy in the global ocean microbiome. Science 353:1272–1277. doi:10.1126/science.aaf4507 27634532

[58] Segata N, Izard J, Waldron L, Gevers D, Miropolsky L, Garrett WS, Huttenhower C. 2011. Metagenomic biomarker discovery and explanation. Genome Biol 12:R60. doi:10.1186/gb-2011-12-6-r60 21702898 PMC3218848

[59] Revelle WR. 2017. psych: procedures for personality and psychological research

[60] Liu Y, Gong L, Mu X, Zhang Z, Zhou T, Zhang S. 2020. Characterization and co-occurrence of microbial community in epiphytic biofilms and surface sediments of wetlands with submersed macrophytes. Sci Total Environ 715:136950. doi:10.1016/j.scitotenv.2020.136950 32007899

[61] Deng Y, Jiang Y-H, Yang Y, He Z, Luo F, Zhou J. 2012. Molecular ecological network analyses. BMC Bioinformatics 13:1–20. doi:10.1186/1471-2105-13-113 22646978 PMC3428680

[62] Banerjee S, Baah-Acheamfour M, Carlyle CN, Bissett A, Richardson AE, Siddique T, Bork EW, Chang SX. 2016. Determinants of bacterial communities in Canadian agroforestry systems. Environ Microbiol 18:1805–1816. doi:10.1111/1462-2920.12986 26184386

[63] Burns AR, Stephens WZ, Stagaman K, Wong S, Rawls JF, Guillemin K, Bohannan BJ. 2016. Contribution of neutral processes to the assembly of gut microbial communities in the zebrafish over host development. ISME J 10:655–664. doi:10.1038/ismej.2015.142 26296066 PMC4817674

[64] Fine PVA, Kembel SW. 2011. Phylogenetic community structure and phylogenetic turnover across space and edaphic gradients in western Amazonian tree communities. Ecography 34:552–565. doi:10.1111/j.1600-0587.2010.06548.x

[65] Tripathi BM, Stegen JC, Kim M, Dong K, Adams JM, Lee YK. 2018. Soil pH mediates the balance between stochastic and deterministic assembly of bacteria. ISME J 12:1072–1083. doi:10.1038/s41396-018-0082-4 29515169 PMC5864241

[66] Wang J, Shen J, Wu Y, Tu C, Soininen J, Stegen JC, He J, Liu X, Zhang L, Zhang E. 2013. Phylogenetic beta diversity in bacterial assemblages across ecosystems: deterministic versus stochastic processes. ISME J 7:1310–1321. doi:10.1038/ismej.2013.30 23446837 PMC3695296

[67] Webb CO, Ackerly DD, Kembel SW. 2008. Phylocom: software for the analysis of phylogenetic community structure and trait evolution. Bioinformatics 24:2098–2100. doi:10.1093/bioinformatics/btn358 18678590

[68] Kembel SW, Eisen JA, Pollard KS, Green JL. 2011. The phylogenetic diversity of metagenomes. PLoS One 6:e23214. doi:10.1371/journal.pone.0023214 21912589 PMC3166145

[69] Chase JM, Kraft NJB, Smith KG, Vellend M, Inouye BD. 2011. Using null models to disentangle variation in community dissimilarity from variation in α-diversity. Ecosphere 2:art24. doi:10.1890/ES10-00117.1

[70] Shang S, Li L, Xiao H, Chen J, Zang Y, Wang J, Tang X. 2023. Studies on the composition and diversity of seagrass ruppia sinensis rhizosphere mmicroorganisms in the Yellow River Delta. Plants (Basel) 12:1435. doi:10.3390/plants12071435 37050062 PMC10097283

[71] Chaffron S, Rehrauer H, Pernthaler J, von Mering C. 2010. A global network of coexisting microbes from environmental and whole-genome sequence data. Genome Res 20:947–959. doi:10.1101/gr.104521.109 20458099 PMC2892096

[72] Wang Y, Zhou P, Zhou W, Huang S, Peng C, Li D, Li G. 2023. Network analysis indicates microbial assemblage differences in life stages of Cladophora. Appl Environ Microbiol 89:e0211222. doi:10.1128/aem.02112-22 36880773 PMC10057885

[73] Watts DJ, Strogatz SH. 1998. Collective dynamics of “small-world” networks. Nature 393:440–442. doi:10.1038/30918 9623998

[74] Halperin RF, Stafford P, Emery JS, Navalkar KA, Johnston SA. 2012. GuiTope: an application for mapping random-sequence peptides to protein sequences. BMC Bioinformatics 13:1. doi:10.1186/1471-2105-13-1 22214541 PMC3280184

[75] Wang X, Wang H, Liang Y, McMinn A, Wang M. 2024. Community organization and network complexity and stability: contrasting strategies of prokaryotic versus eukaryotic microbiomes in the Bohai Sea and Yellow Sea. mSphere 9:e0039524. doi:10.1128/msphere.00395-24 39136485 PMC11423591

[76] Wood DC, Hayasaka SS. 1981. Chemotaxis of rhizoplane bacteria to amino acids comprising eelgrass (Zostera marina L.) root exudate. J Exp Mar Biol Ecol 50:153–161. doi:10.1016/0022-0981(81)90047-2

[77] Perry C, Dennison W. 1999. Microbial nutrient cycling in seagrass sediments. AGSO J Aust Geol Geophys 17:227–232.

[78] Nielsen LB, Finster K, Welsh DT, Donelly A, Herbert RA, de Wit R, Lomstein BA. 2001. Sulphate reduction and nitrogen fixation rates associated with roots, rhizomes and sediments from Zostera noltii and Spartina maritima meadows. Environ Microbiol 3:63–71. doi:10.1046/j.1462-2920.2001.00160.x 11225724

[79] Kurilenko VV, Ivanova EP, Mikhaĭlov VV. 2007. Peculiarities of adhesion of epiphytic bacteria on leaves of the seagrass Zostera marina and on abiotic surfaces. Microbiology (Reading, Engl) 76:442–445. doi:10.1134/S0026261707040091 17974207

[80] Sun F, Zhang X, Zhang Q, Liu F, Zhang J, Gong J. 2015. Seagrass (Zostera marina) colonization promotes the accumulation of diazotrophic bacteria and alters the relative abundances of specific bacterial lineages involved in benthic carbon and sulfur cycling. Appl Environ Microbiol 81:6901–6914. doi:10.1128/AEM.01382-15 26209674 PMC4561699

[81] Kurtz JC, Yates DF, Macauley JM, Quarles RL, Genthner FJ, Chancy CA, Devereux R. 2003. Effects of light reduction on growth of the submerged macrophyte Vallisneria americana and the community of root-associated heterotrophic bacteria. J Exp Mar Biol Ecol 291:199–218. doi:10.1016/S0022-0981(03)00120-5

[82] Cúcio C, Engelen AH, Costa R, Muyzer G. 2016. Rhizosphere microbiomes of European seagrasses are selected by the plant, but are not species specific. Front Microbiol 7:440. doi:10.3389/fmicb.2016.00440 27065991 PMC4815253

[83] Ettinger CL, Voerman SE, Lang JM, Stachowicz JJ, Eisen JA. 2017. Microbial communities in sediment from Zostera marina patches, but not the Z. marina leaf or root microbiomes, vary in relation to distance from patch edge. PeerJ 5:e3246. doi:10.7717/peerj.3246 28462046 PMC5410140

[84] Turzynski V, Griesdorn L, Moraru C, Soares AR, Simon SA, Stach TL, Rahlff J, Esser SP, Probst AJ. 2023. Virus-host dynamics in archaeal groundwater biofilms and the associated bacterial community composition. Viruses 15:910. doi:10.3390/v15040910 37112890 PMC10143303

[85] Choi A, Lee TK, Cho H, Lee W-C, Hyun J-H. 2022. Shifts in benthic bacterial communities associated with farming stages and a microbiological proxy for assessing sulfidic sediment conditions at fish farms. Mar Pollut Bull 178:113603. doi:10.1016/j.marpolbul.2022.113603 35390629

[86] Thomas F, Giblin AE, Cardon ZG, Sievert SM. 2014. Rhizosphere heterogeneity shapes abundance and activity of sulfur-oxidizing bacteria in vegetated salt marsh sediments. Front Microbiol 5:309. doi:10.3389/fmicb.2014.00309 25009538 PMC4068000

[87] Martin BC, Bougoure J, Ryan MH, Bennett WW, Colmer TD, Joyce NK, Olsen YS, Kendrick GA. 2019. Oxygen loss from seagrass roots coincides with colonisation of sulphide-oxidising cable bacteria and reduces sulphide stress. ISME J 13:707–719. doi:10.1038/s41396-018-0308-5 30353038 PMC6461758

[88] Wang L, Tomas F, Mueller RS. 2020. Nutrient enrichment increases size of Zostera marina shoots and enriches for sulfur and nitrogen cycling bacteria in root-associated microbiomes. FEMS Microbiol Ecol 96:fiaa129. doi:10.1093/femsec/fiaa129 32578844

[89] Peng X, Zhang X, Li Z, Zhang S, Zhang X, Zhang H, Lin Q, Li X, Zhang L, Ge F, Wu Z, Liu B. 2024. Unraveling the ecological mechanisms of aluminum on microbial community succession in epiphytic biofilms on Vallisneria natans leaves: novel insights from microbial interactions. J Hazard Mater 469:133932. doi:10.1016/j.jhazmat.2024.133932 38484659

[90] Shade A, Kent AD, Jones SE, Newton RJ, Triplett EW, McMahon KD. 2007. Interannual dynamics and phenology of bacterial communities in a eutrophic lake. Limnol Ocean 52:487–494. doi:10.4319/lo.2007.52.2.0487

[91] Li C, Liu J, Chen X, Ren H, Su B, Ma K, Tu Q. 2022. Determinism governs the succession of disturbed bacterioplankton communities in a coastal maricultural ecosystem. Sci Total Environ 828:154457. doi:10.1016/j.scitotenv.2022.154457 35278542

[92] Galván V, Pascutti F, Sandoval NE, Lanfranconi MP, Lozada M, Arabolaza AL, Mac Cormack WP, Alvarez HM, Gramajo HC, Dionisi HM. 2023. High wax ester and triacylglycerol biosynthesis potential in coastal sediments of Antarctic and Subantarctic environments. PLoS One 18:e0288509. doi:10.1371/journal.pone.0288509 37459319 PMC10351704

[93] Zhang Y, Wang T, Sun H, Shen P. 2024. Microbial community structure and function in rhizosphere of phyllospadix iwatensis on shandong coast. J Yant Uni 37:26–36. doi:10.13951/j.cnki.37-1213/n.230402

[94] Liu P, Zou S, Zhang H, Liu Q, Song Z, Huang Y, Hu X. 2023. Genome-resolved metagenomics provides insights into the microbial-mediated sulfur and nitrogen cycling in temperate seagrass meadows. Front Mar Sci 10. doi:10.3389/fmars.2023.1245288

[95] Zhang F, Zhang W, Qian D-K, Dai K, van Loosdrecht MCM, Zeng RJ. 2019. Synergetic alginate conversion by a microbial consortium of hydrolytic bacteria and methanogens. Water Res 163:114892. doi:10.1016/j.watres.2019.114892 31351355

[96] Wang B, Zheng X, Zhang H, Yu X, Lian Y, Yang X, Yu H, Hu R, He Z, Xiao F, Yan Q. 2021. Metagenomic insights into the effects of submerged plants on functional potential of microbial communities in wetland sediments. Mar Life Sci Technol 3:405–415. doi:10.1007/s42995-021-00100-3 37073260 PMC10077182

[97] Salam N, Xian W-D, Asem MD, Xiao M, Li W-J. 2021. From ecophysiology to cultivation methodology: filling the knowledge gap between uncultured and cultured microbes. Mar Life Sci Technol 3:132–147. doi:10.1007/s42995-020-00064-w 37073336 PMC10077289

[98] Ke-chang N, Yi-ning L, Ze-hao S, Fang-liang H, Jing-yun F. 2009. Community assembly: the relative importance of neutral theory and niche theory. Biodiver Sci 17:579. doi:10.3724/SP.J.1003.2009.09142

[99] Price JN, Gazol A, Tamme R, Hiiesalu I, Pärtel M. 2014. The functional assembly of experimental grasslands in relation to fertility and resource heterogeneity. Funct Ecol 28:509–519. doi:10.1111/1365-2435.12186

[100] Gazol A, Tamme R, Price JN, Hiiesalu I, Laanisto L, Pärtel M. 2013. A negative heterogeneity-diversity relationship found in experimental grassland communities. Oecologia 173:545–555. doi:10.1007/s00442-013-2623-x 23468237

[101] Zhang X, Wu Y, Liu S, Li J, Jiang Z, Luo H, Huang X. 2024. Plant growth and development of tropical seagrass determined rhizodeposition and its related microbial community. Mar Pollut Bull 199:115940. doi:10.1016/j.marpolbul.2023.115940 38150979

[102] Blagodatskaya EV, Blagodatsky SA, Anderson T-H, Kuzyakov Y. 2007. Priming effects in Chernozem induced by glucose and N in relation to microbial growth strategies. Agric, Ecosyst Environ, Appl Soil Ecol 37:95–105. doi:10.1016/j.apsoil.2007.05.002

[103] Hicks LC, Lajtha K, Rousk J. 2021. Nutrient limitation may induce microbial mining for resources from persistent soil organic matter. Ecology 102:e03328. doi:10.1002/ecy.3328 33705567

