# Supplementary figures and images for: Exploring the structure and assembly of seagrass microbial communities in rhizosphere and phyllosphere
Source: Appl Environ Microbiol. 2025 Feb 24;91(3):e02437-24. doi: 10.1128/aem.02437-24 (PMC11921323; doi:10.1128/aem.02437-24)

A

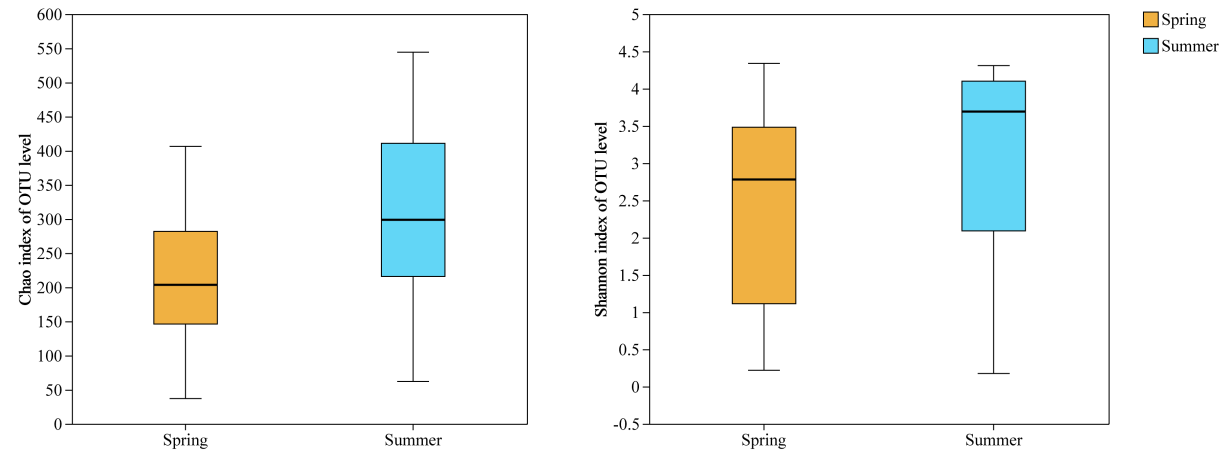

B

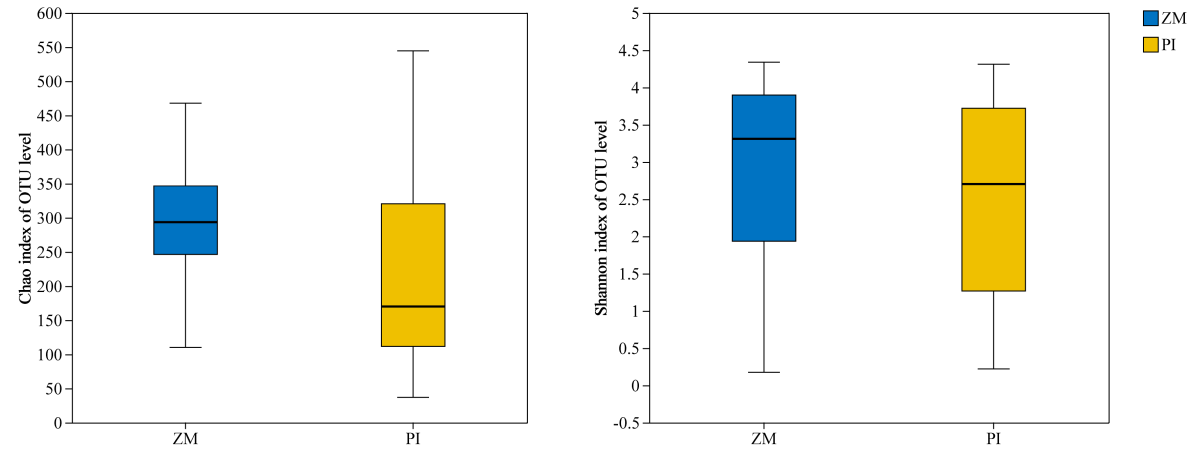

C

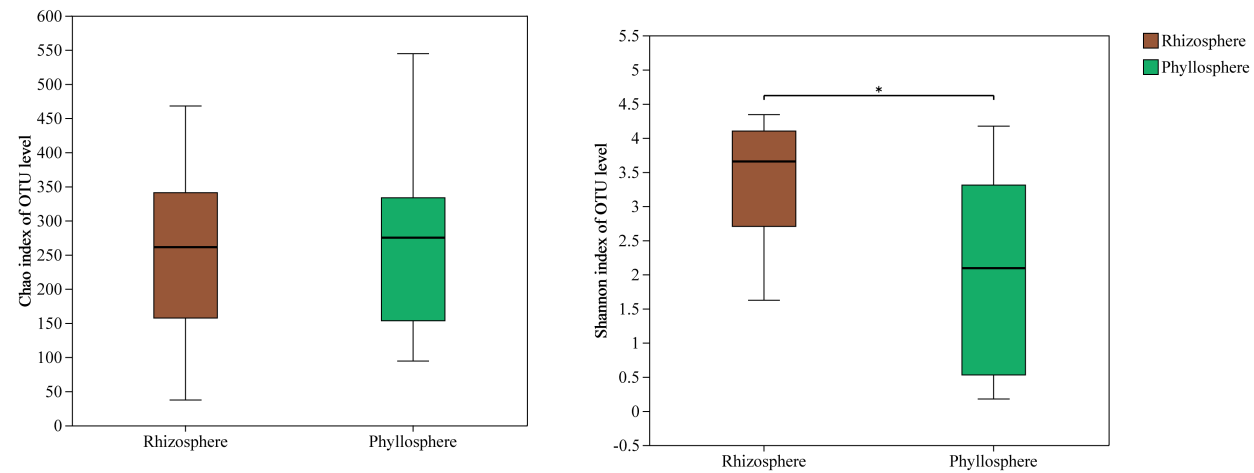

Supplement: Fig. S1 — Shannon and Chao diversity of fungal communities in two periods of seagrass, in two seagrass species, and between rhizosphere and phyllosphere. [file aem.02437-24-s0001.pdf]

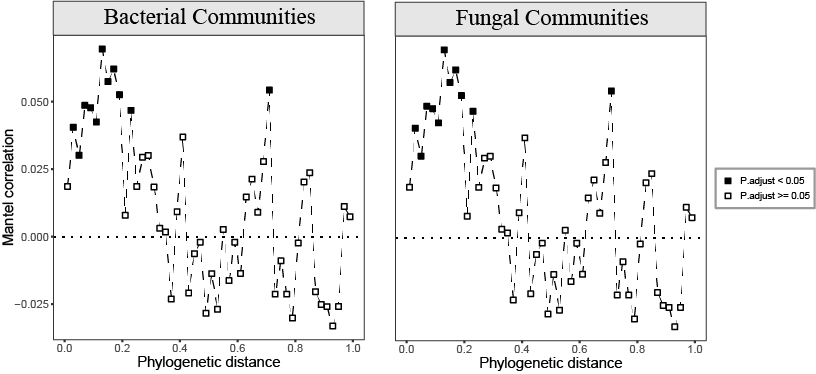

Supplement: Fig. S3 — Mantel correlograms between the pairwise of OTU niche distances and phylogenetic distances with 999 permutations (Spearman correlation) in bacterial communities and fungal communities. [file aem.02437-24-s0003.tif]

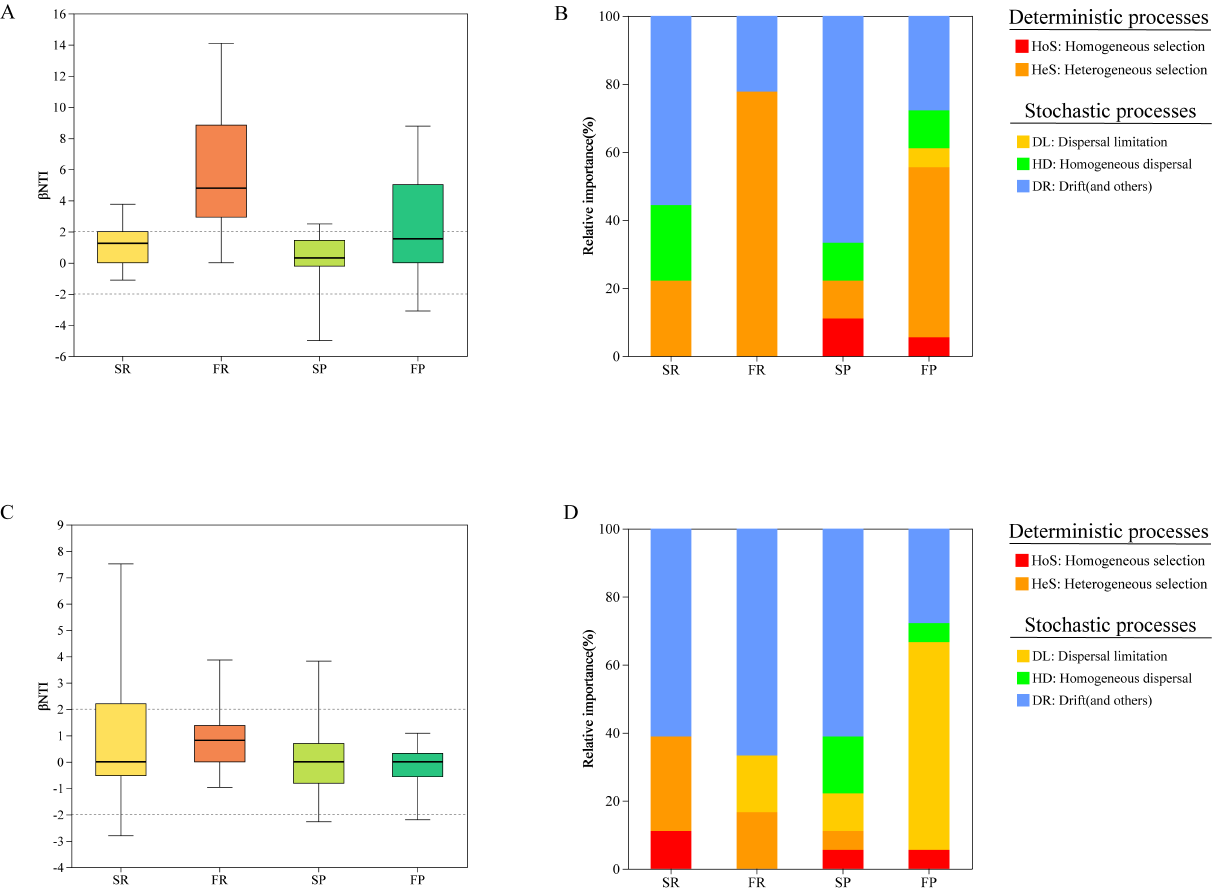

Supplement: Fig. S4 — Evaluation of the assembly mechanisms of bacterial communities and fungal communities in the SR, FR, SP, and FP by using null model analysis. [file aem.02437-24-s0004.tif]
