# Supplementary material for: Exploring the structure and assembly of seagrass microbial communities in rhizosphere and phyllosphere
Source: Appl Environ Microbiol. 2025 Feb 24;91(3):e02437-24. doi: 10.1128/aem.02437-24 (PMC11921323; doi:10.1128/aem.02437-24)
Supplement: Fig. S2 — Co-occurrence network and keystone taxa of fungal communities at the OTU levels in rhizosphere and phyllosphere samples. [file aem.02437-24-s0002.pdf]

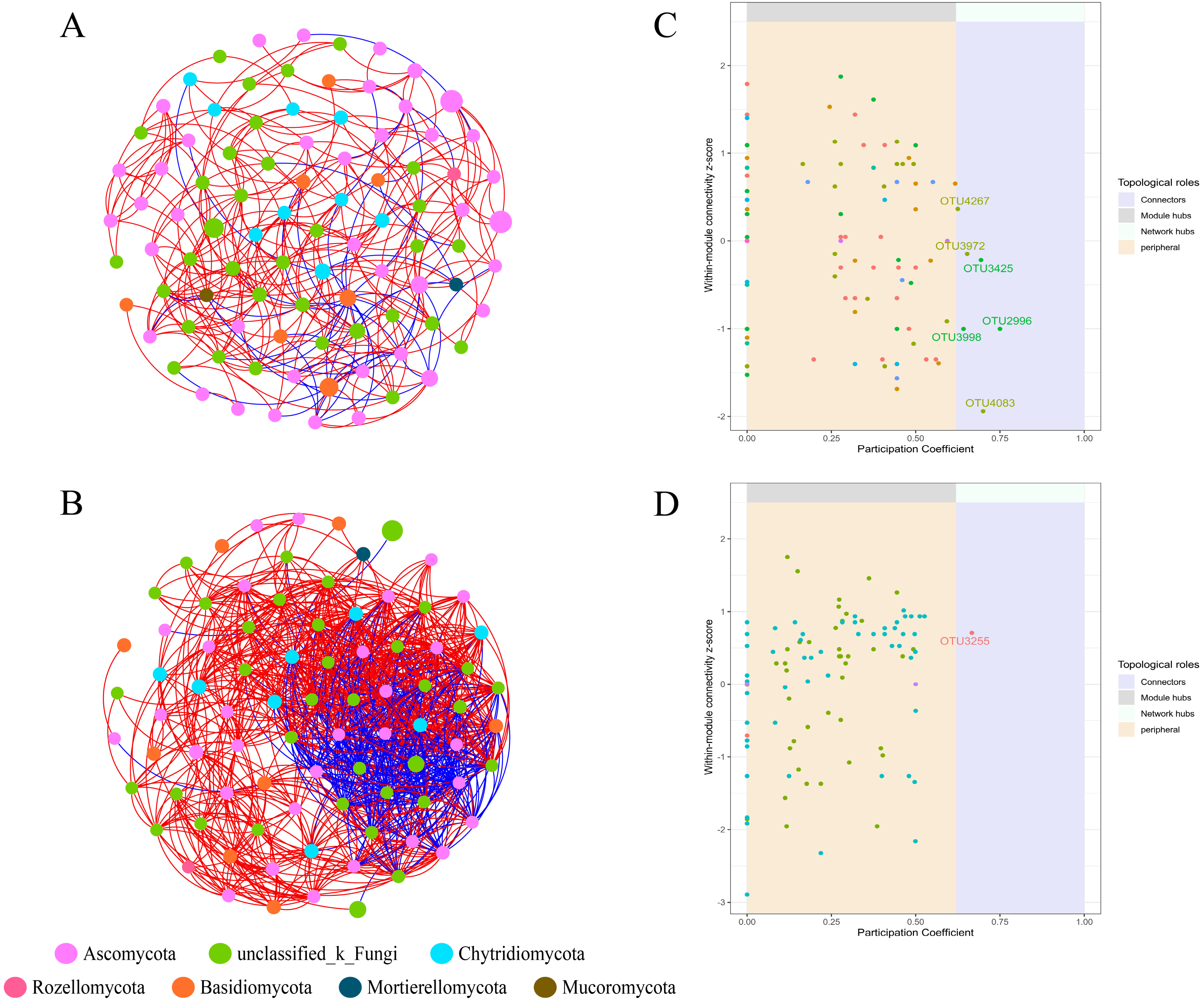

A
B
C
D
Ascomycota
unclassified_k_Fungi
Chytridiomycota
Rozellomycota
Basidiomycota
Mortierellomycota
Mucoromycota
Within-module connectivity z-score
Participation Coefficient
0.00
0.25
0.50
0.75
1.00
2
1
0
-1
-2
-3
OTU4267
OTU3972
OTU3425
OTU2996
OTU3998
OTU4083
OTU3255
Topological roles
Connectors
Module hubs
Network hubs
peripheral
