# Supplementary material for: Exploring the structure and assembly of seagrass microbial communities in rhizosphere and phyllosphere
Source: Appl Environ Microbiol. 2025 Feb 24;91(3):e02437-24. doi: 10.1128/aem.02437-24 (PMC11921323; doi:10.1128/aem.02437-24)
Supplement: Supplemental legends — Legends for Fig. S1 to S4 and Tables S1 to S4. [file aem.02437-24-s0005.docx]

**Supplemental Material Legends**

**Figure S1.** (A) Shannon and Chao diversity of fungal communities in two periods of seagrass. (B) Shannon and Chao diversity of fungal communities in two seagrass species, where ZM is *Zostera marina* and PI is *Phyllospadix iwatensis*. (C) Shannon and Chao diversity of the fungal communities between rhizosphere and phyllosphere. Boxplot showing 12 samples (n=12). Statistical comparisons of the data were conducted using the Wilcoxon rank-sum test. Significance levels were denoted as follows: “*” for *P* < 0.05, “**” for *P* < 0.01, and “***” for *P* < 0.001.

**Figure S2.** Co-occurrence network and Keystone taxa of fungal communities at the OTU levels in rhizosphere samples (A), (C) and phyllosphere samples (B), (D). The top 100 OTUs for relative abundance were selected for each sample. Nodes in the network are color-coded for different fungal phyla, while lines connecting nodes represent correlations between OTUs. Positive correlations are indicated by red lines and negative ones by blue lines. Specifically, module hubs were defined as taxa with Zi ≥ 2.5 and Pi < 0.62, connectors as taxa with Zi < 2.5 and Pi ≥ 0.62, and network hubs as taxa with Zi ≥ 2.5 and Pi ≥ 0.62.

**Figure S3.** Mantel correlograms between the pairwise of OTU niche distances and phylogenetic distances with 999 permutations (Spearman correlation) in bacterial communities and fungal communities. Phylogenetic distance was standardized to range from 0 to 1. Black points represent significant phylogenetic signals at the significance level of α < 0.05 after Bonferroni multiple testing.

**Figure S4.** Evaluation of the assembly mechanisms of bacterial communities and fungal communities in the SR, FR, SP and FP by using null model analysis. The contributions of deterministic processes (|βNTI| ≥ 2) and random processes (|βNTI| < 2) to the assembly of bacterial communities (A) and fungal communities (C) assembly in the SR, FR, SP and FP. The relative contributions of different ecological processes driving the assembly of bacterial communities (B) and fungal communities (D) in the SR, FR, SP and FP.

**Supplementary Table 1.** Analysis of different time periods, microhabitats and seagrass species explaining bacterial community structure (PERMANOVA based on Bray-Curtis distance).

**Supplementary Table 2.** Analysis of different time periods, microhabitats and seagrass species explaining fungal community structure (PERMANOVA based on Bray-Curtis distance).

**Supplementary Table 3.** The properties of co-occurrence networks of bacterial and fungal communities in the rhizosphere and phyllosphere.

**Supplementary Table 4.** List of keystone nodes and their taxonomic information in rhizosphere and phyllosphere.
