# Supplementary material for: Exploring the structure and assembly of seagrass microbial communities in rhizosphere and phyllosphere
Source: Appl Environ Microbiol. 2025 Feb 24;91(3):e02437-24. doi: 10.1128/aem.02437-24 (PMC11921323; doi:10.1128/aem.02437-24)
Supplement: Table S1 — Analysis of different time periods, microhabitats, and seagrass species explaining bacterial community structure (PERMANOVA based on Bray-Curtis distance). [file aem.02437-24-s0006.docx]

|  | Df | Sums of squares | Mean squares | F.Model | Variation (R2) | Pr (>F) |
| --- | --- | --- | --- | --- | --- | --- |
| Species | 1 | 0.345580916 | 0.345580916 | 2.41805996 | 0.053619347 | 0.042 |
| Time | 1 | 0.581262201 | 0.581262201 | 4.067142571 | 0.090186981 | 0.008 |
| Microhabitats | 1 | 2.659903636 | 2.659903636 | 18.61157891 | 0.412703042 | 0.001 |
| Residuals | 20 | 2.858332062 | 0.142916603 |  | 0.44349063 |  |
| Total | 23 | 6.445078814 |  |  | 1 |  |
