# Supplementary material for: Exploring the structure and assembly of seagrass microbial communities in rhizosphere and phyllosphere
Source: Appl Environ Microbiol. 2025 Feb 24;91(3):e02437-24. doi: 10.1128/aem.02437-24 (PMC11921323; doi:10.1128/aem.02437-24)
Supplement: Table S2 — Analysis of different time periods, microhabitats, and seagrass species explaining fungal community structure (PERMANOVA based on Bray-Curtis distance). [file aem.02437-24-s0007.docx]

|  | Df | Sums of squares | Mean squares | F.Model | Variation (R2) | Pr(>F) |
| --- | --- | --- | --- | --- | --- | --- |
| Species | 1 | 0.85385 | 0.85385 | 2.9268 | 0.09564 | 0.001 |
| Time | 1 | 1.05664 | 1.05664 | 3.62192 | 0.11835 | 0.001 |
| Microhabitats | 1 | 1.18255 | 1.18255 | 4.05352 | 0.13246 | 0.001 |
| Residuals | 20 | 5.8347 | 0.29174 | 0 | 0.65355 | 0 |
| Total | 23 | 8.92775 | 0 | 0 | 1 | 0 |
