# Supplementary material for: Exploring the structure and assembly of seagrass microbial communities in rhizosphere and phyllosphere
Source: Appl Environ Microbiol. 2025 Feb 24;91(3):e02437-24. doi: 10.1128/aem.02437-24 (PMC11921323; doi:10.1128/aem.02437-24)
Supplement: Table S3 — Properties of co-occurrence networks of bacterial and fungal communities in the rhizosphere and phyllosphere. [file aem.02437-24-s0008.docx]

| **Communities** | **Properties** | **Rhizosphere** | **Phyllosphere** |
| --- | --- | --- | --- |
| Bacterial community | Nodes | 97 | 100 |
|  | Edges | 895 | 1460 |
|  | Modularity | 0.354 | 0.403 |
|  | Network density | 0.192 | 0.295 |
|  | Average path length | 2.23 | 1.852 |
|  | Average clustering coefficient | 0.596 | 0.663 |
|  | Positive proportions | 79.9% | 66.4% |
|  | Negative proportions | 20.1% | 33.6% |
| Fungal community | Nodes | 91 | 83 |
|  | Edges | 326 | 1017 |
|  | Modularity | 0.5 | 0.28 |
|  | Network density | 0.08 | 0.299 |
|  | Average path length | 2.909 | 2.011 |
|  | Average clustering coefficient | 0.419 | 0.669 |
|  | Positive proportions | 83.7% | 75.5% |
|  | Negative proportions | 16.3% | 24.5% |
