# Supplementary material for: Exploring the structure and assembly of seagrass microbial communities in rhizosphere and phyllosphere
Source: Appl Environ Microbiol. 2025 Feb 24;91(3):e02437-24. doi: 10.1128/aem.02437-24 (PMC11921323; doi:10.1128/aem.02437-24)
Supplement: Table S4 — Keystone nodes and their taxonomic information in rhizosphere and phyllosphere. [file aem.02437-24-s0009.docx]

| **Network** | **OTU number** | **Role** | **Domain** | **Phylum** | **Class** | **Order** | **Family** | **Genus** |  |
| --- | --- | --- | --- | --- | --- | --- | --- | --- | --- |
| Rhizosphere | OTU5006 | Connector | Bacteria | Proteobacteria | Alphaproteobacteria | Rhodobacterales | Rhodobacteraceae | *Pseudoruegeria* |  |
|  | OTU6175 | Connector | Bacteria | Actinobacteriota | Acidimicrobiia | Microtrichales | Ilumatobacteraceae | *Ilumatobacter* |  |
|  | OTU9058 | Connector | Bacteria | Acidobacteriota | Vicinamibacteria | Subgroup_17 | norank_Subgroup_17 | *norank_norank_Subgroup_17* |  |
|  | OTU4267 | Connector | Eukaryota | Unclassified | Unclassified | Unclassified | Unclassified | *Unclassified* |  |
|  | OTU3972 | Connector | Eukaryota | Unclassified | Unclassified | Unclassified | Unclassified | *Unclassified* |  |
|  | OTU4083 | Connector | Eukaryota | Ascomycota | Unclassified | Unclassified | Unclassified | *Unclassified* |  |
|  | OTU3998 | Connector | Eukaryota | Chytridiomycota | Lobulomycetes | Lobulomycetales | Lobulomycetaceae | *Clydaea* |  |
|  | OTU2996 | Connector | Eukaryota | Unclassified | Unclassified | Unclassified | Unclassified | *Unclassified* |  |
|  | OTU3425 | Connector | Eukaryota | Unclassified | Unclassified | Unclassified | Unclassified | *Unclassified* |  |
| Phyllosphere | OTU1641 | Connector | Bacteria | Bacteroidota | Bacteroidia | Flavobacteriales | Flavobacteriaceae | *Algibacter* | *Algibacter_lectus* |
|  | OTU3255 | Connector | Eukaryota | Ascomycota | Sordariomycetes | Unclassified | Unclassified | *Unclassified* |  |
